# Urokinase-type plasminogen activator receptor (uPAR) assessed by liquid biopsies and PET/CT for prognostication in head and neck cancer patients

**DOI:** 10.1038/s41598-022-21175-7

**Published:** 2022-11-09

**Authors:** Louise Madeleine Risør, Tina Binderup, Marie Øbro Fosbøl, Kim Francis Andersen, Annika Loft, Jeppe Friborg, Andreas Kjaer

**Affiliations:** 1 Department of Clinical Physiology and Nuclear Medicine & Cluster for Molecular Imaging, Copenhagen University Hospital, Rigshospitalet, Blegdamsvej 9, 2100 Copenhagen, Denmark; 2 Department of Biomedical Sciences, University of Copenhagen, Copenhagen, Denmark; 3 Department of Clinical Oncology, Copenhagen University Hospital, Rigshospitalet, Copenhagen, Denmark

**Keywords:** Biomarkers, Oncology

## Abstract

Strong prognostic biomarkers are lacking regarding the stratification of treatment and surveillance regimens in head and neck squamous cell carcinoma (HNSCC). The study aimed to assess the prognostic value of soluble urokinase-type plasminogen activator receptor in plasma (suPAR) compared to evaluation by uPAR-positron-emission-tomography (PET) in HNSCC patients. Plasma from 19 controls and 49 HNSCC patients referred to curatively intended radiotherapy (2017–2021) was collected pre-treatment and post-treatment (n = 37). Information on uPAR-PET was available from previous evaluation. Patient median suPAR was significantly higher pre- and post-treatment compared to controls (p = 0.013, p = 0.003) and increased significantly during radiotherapy (p = 0.003). Pre-treatment suPAR did not predict survival outcomes. Post-treatment suPAR significantly predicted RFS (HR = 6.67 (95% CI 1.44–30.9) p = 0.015), but not OS (HR = 3.29 (95% CI 0.882–12.3) p = 0.076) in univariate analysis. RFS prediction was maintained for post-treatment suPAR in multivariate analysis, including TNM-stage (HR = 6.62 (95% CI 1.40–31.4) p = 0.017). Pre-treatment uPAR-PET/CT and post-treatment suPAR was available in 24 patients. High uPAR-estimates on both modalities was significantly associated with poor RFS compared to patients with low uPAR-estimates (log-rank, p = 0.008). Patients with discordant uPAR-estimates (one-low/one-high) were at intermediate risk, although non-significant (p = 0.131). In conclusion, pre-treatment suPAR did not predict RFS or OS. Pre-treatment uPAR-PET and post-treatment suPAR predicted RFS.

## Introduction

Head and Neck Squamous Cell Carcinoma (HNSCC) is traditionally caused by tobacco and alcohol, but in the past decades Human Papilloma Virus (HPV) has been associated with an increasing incidence of oropharyngeal cancers^1^. Despite improvements in multimodality treatment options, HPV-negative HNSCC still has a poor prognosis due to loco-regional recurrence and distant metastasis^1,2^.

Besides p16 no prognostic biomarkers have been applied in clinical practice, but considerable effort has been invested in the discovery of new prognostic markers in HNSCC to tailor treatment and surveillance strategy according to individual risk profiles^2^.

The urokinase-type Plasminogen Activator Receptor (uPAR) is a membrane-bound receptor and a part of the proteolytic cascade. Upon binding to its ligand urokinase-type Plasminogen Activator (uPA) promotes the activation of plasmin^3^. Plasmin is a key enzyme involved in the degradation of extracellular matrix proteins, tissue remodeling and cell migration, which are essential processes in cancer invasion and metastasis. uPAR can be shed from the cell surface and the soluble forms of uPAR (suPAR) and cleaved subdomains have been identified in body fluids^3,4^. Several studies have documented the total amount of all uPAR forms to be strong prognostic markers in different types of cancers both in tissue and liquid biopsies^5–9^. Furthermore, increasing uPAR levels during radiotherapy have been associated with poor prognosis in tissue and liquid biopsies^7,8,10^.

Currently, invasive biopsies are required for histology, staging and p16-status determination. However, biopsies only represent a fraction of an otherwise heterogenous cancer at baseline. Liquid biopsies circumvent tissue sampling bias and offers a non-invasive whole-tumor characterization that can be repeated to follow the current status of the cancer cells (evolving subclones)^11^. However, several studies report that plasma uPAR and liquid biopsies in general have the disadvantage of not being able to distinguish between the origin of the biomarker^12,13^. Consequently, plasma uPAR may miss the regional variations important for uPAR evaluation, i.e. a high expression from a limited tumor volume and a low expression from a larger volume cannot be differentiated.

In contrast, uPAR-positron emission tomography (PET/CT) using the tracer [^68^Ga]Ga-NOTA-AE105 is a non-invasive alternative to evaluate the regional uPAR-expression in the entire tumor volume without the risk of sampling bias and extra-tumoral origin, and has demonstrated strong prognostic value in HNSCC^14^. However, as blood sampling carries a fraction of the cost of uPAR-PET/CT, the question arises how uPAR measured in blood compares to uPAR-PET regarding prognostic value.

The aim of the current study was therefore to investigate the prognostic value of liquid biopsies monitoring plasma uPAR pre- and post-treatment in HNSCC patients referred to curatively intended radiotherapy and to compare them with the prognostic information obtained by pre-treatment uPAR-PET/CT.

## Results

### Patients

In total, 19 healthy controls and 49 patients recently diagnosed with HNSCC in the pharynx, larynx or oral cavity and referred to curatively intended radiotherapy at Rigshospitalet or Næstved Hospital, contributed with one or more blood samples to the biobank.

A total of 37 pre-treatment- and 37 2-months post-treatment samples were donated from the 49 patients. In 26 patients, both a pre- and post-treatment sample was available.

Patient and control characteristics are shown in Table 1. The patients were predominantly male (85.7%) smokers (mean pack years; 32.5 years), and more than half the patients (57.2%) presented with early stage disease (Stage 1–2) with no primary regional nodal disease in 38.8% of the cases. Most of the primary tumors were located in the oropharynx (71.4%) of which 82.9% were p16 positive.

**Table 1:** Patient and control characteristics. Performance status (PS) according to the WHO classification. Epstein–Barr virus (EBV). Tumor (T). Node (N).

Characteristics	Patients		Controls		p value
	n	%	n	%	
**Total**	49	100	19	100	
**Gender**					
Male	42	85.7	11	57.9	0.022
Female	7	14.3	8	42.1	
**Age**					
Mean	64.3		65.2		0.662
Range	49–84		55–79		
**PS**					
0	36	73.5	19	100	0.014
1	13	26.5	0	0	
**Smoking**					
Never smokers	5	10.2	9	47.4	0.018 (never/former)
Former smokers	22	44.9	7	36.8	0.309 (former/current)
Current smokers	22	44.9	3	15.8	0.002 (never/current)
Pack years; mean	32.5		8.79		
Pack years; range	(range 0–150)		(range 0–37.5)		
**Primary site**					
Oral Cavity	1	2.0			
Pharynx					
Rhinopharynx	1	2.0			
Oropharynx	35	71.4			
Hypopharynx	7	14.3			
Larynx	5	10.2			
**P16 (oropharynx)**					
p16 positive	29	(82.9)			
p16 negative	6	(17.1)			
**EBV positive**	1	2.0			
**Stage**					
I	16	32.7			
II	12	24.5			
III	11	22.4			
IV	10	20.4			
**T classification**					
T1	3	6.0			
T2	25	51.0			
T3	11	22.4			
T4	10	20.4			
**N classification**					
N0	14	28.6			
N1	19	38.8			
N2	16	32.7			
**Chemotherapy**					
No cisplatin	18	36.7			
Cisplatin	31	63.3			
**Nimorazole**					
No	8	16.3			
Yes	41	83.7			
**Recurrence**					
Yes	14	28.6			
No	35	71.4			
**Death**					
Yes	11	22.4			
No	38	77.6			

The controls consisted of 11 male (57.9%) and eight (42.1%) female participants with a mean age of 65.2 years (range 55–79 years). Nine were never smokers, seven former smokers and three were current smokers. The mean smoking history was 8.8 pack years (range 0–37.5 years).

### Clinical follow-up

Fourteen patients (28.6%) were diagnosed with a recurrence; nine (18.4%) relapsed at the primary site, two (4.08%) at the primary site and lymph nodes and one (2.04%) in the lymph nodes only. Two (4.08%) were diagnosed with distant metastases to the lungs. In all 14 patients the recurrence was histologically verified. 6/14 (42.9%) of all the recurrences were originally tested p16 positive. Four (66%) p16 recurrences relapsed loco-regionally and two (33.3%) with distant metastasis. All 14 patients who experienced a relapse completed all fractions of the primary radiotherapy. Among the patients diagnosed with recurrence, the median time to the recurrence was 8.5 months (range 2.5–35 months); 5.9 months (range 2.5–34) in the p16 negative and 19.0 months (range 4–35) in the p16 positive group.

Throughout the follow-up period 11 patients (22.4%) died; eight (16.3%) due to HNSCC and three (6.1%) due to other causes; one (2.04%) died due to lung cancer, one suffered a violent death and one died of Chronic Obstructive Pulmonary Disease (COPD). None of the non-cancerous deaths were suspicious of recurrence at the previous follow-up or contact with the health care system.

The median follow-up time was 35 months (range 2.5–50 months) for the samples collected pretreatment. All patients were alive at the 2 months routine follow-up. None of the samples were missing due to patients having a recurrence prior to the 2 months follow-up. No patients were lost to follow-up.

### Plasma uPAR concentrations pre- and post-treatment

**Figure 1:**
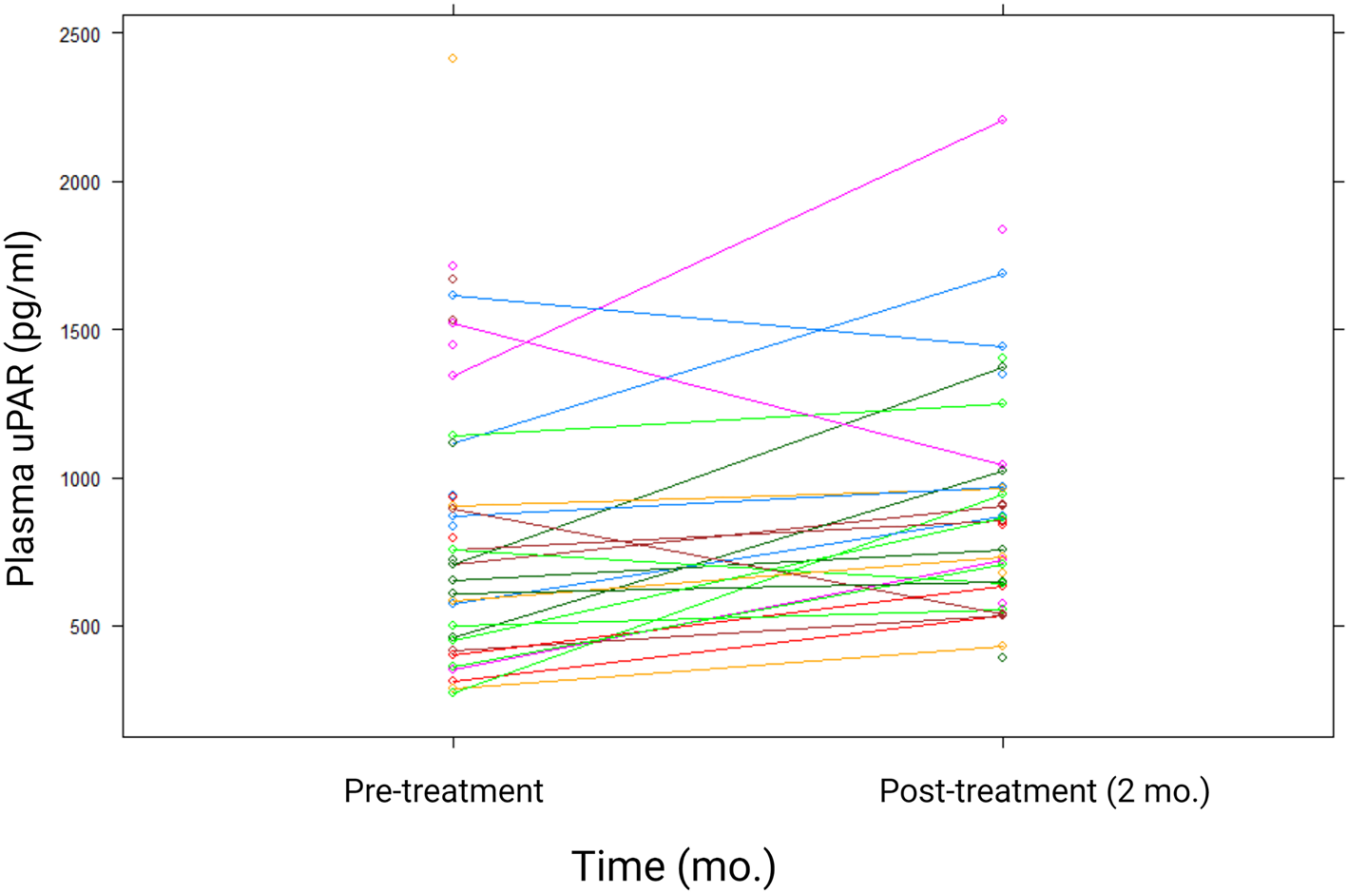
Plasma uPAR development during radiotherapy. Development in plasma uPAR levels in blood samples collected before and 2 months after curatively intended radiotherapy in head and neck squamous cell carcinoma patients.

No samples had uPAR levels below the detection limit of the assay and all were included in the statistical analysis. The development in plasma uPAR concentration over time is shown in Fig. 1, Table 2 for patients and controls. The mean plasma uPAR concentration was 883 pg/ml (S.E.M 80.9) pre-treatment and 925 pg/ml (S.E.M. 66.2) 2-months post-treatment. The control plasma uPAR level was 577 pg/ml (56.5 S.E.M).

**Table 2:** Plasma uPAR levels. Plasma uPAR concentration in pg/ml pre-treatment (0 months) and 2-months post-treatment. Percentile (pct).

	Time	n	Mean	S.E.M	Median	25th pct	75th pct	p-value
Patients	0 mo	37	882.68	80.88	757.4	479.63	1126.27	p = 0.003 (0–2 mo.)
	2 mo	37	924.82	66.15	853.6	642.65	1031.06	
Controls		19	576.83	56.52	496.11	443.58	718.99	p = 0.013 (0 mo.) p = 0.003 (2 mo.)

The uPAR level significantly increased during treatment (p = 0.003, n = 26 in paired analysis). Compared to the controls, the uPAR concentration was significantly elevated in both the pre-treatment and 2-months post-treatment samples (p = 0.013 and p = 0.003).

Pre-treatment plasma uPAR was compared to clinicopathological characteristics and showed a significantly higher level in patients with a smoking history of more than 30 pack years (p = 0.006) and in patients with non-oropharyngeal cancers (p = 0.004). There was no statistically significant association with p16 status, age, gender, or T- and N-stage. Information on all clinicopathological characteristics available of all 49 patients were included in the analysis to increase the statistical power.

### Survival analysis

#### Pre-treatment samples

**Figure 2:**
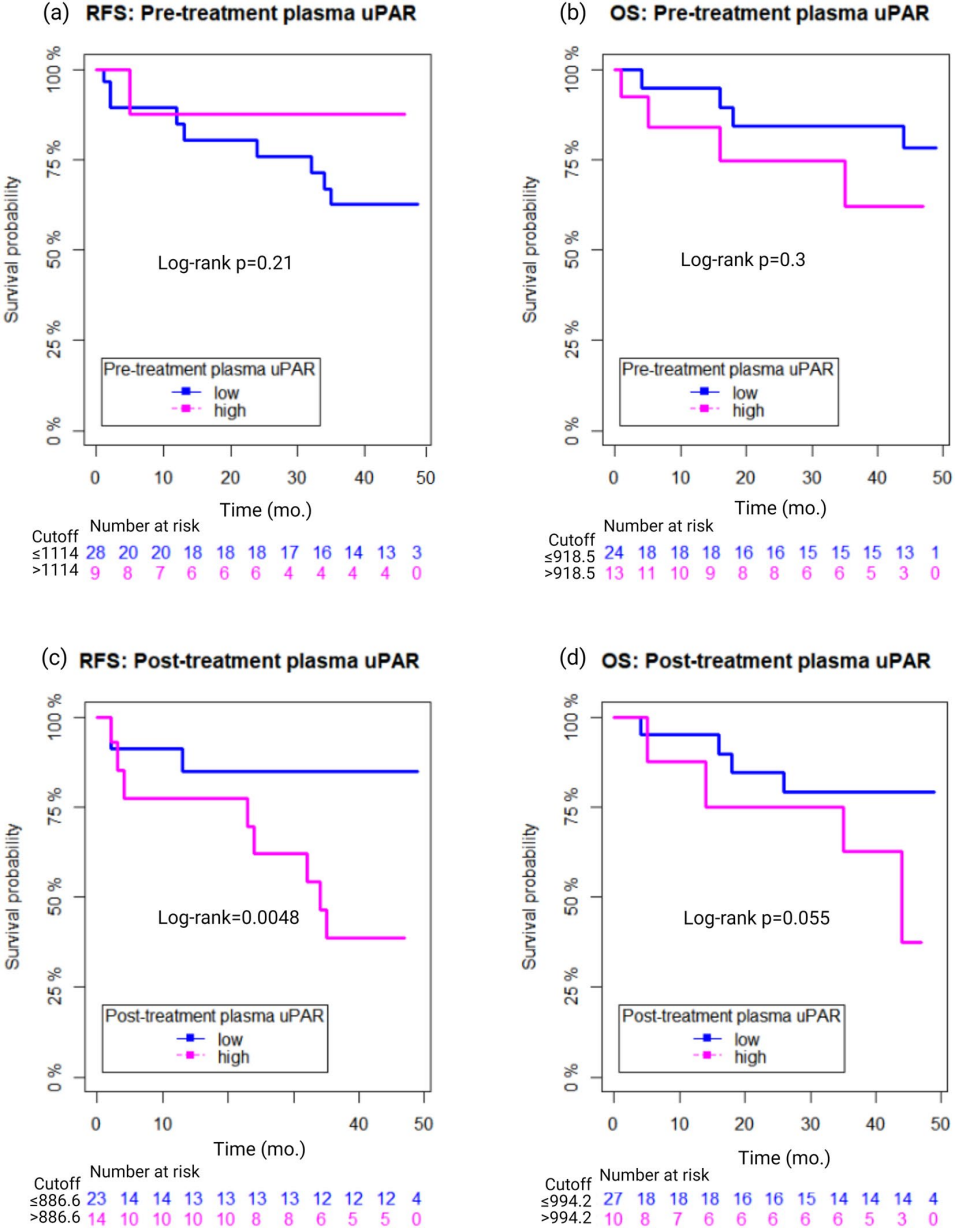
Survival outcomes in head- and neck cancer patients according to plasma uPAR levels pre- and post-radiotherapy treatment. Relapse-free- (RFS) and overall survival (OS) for plasma uPAR measured pre-treatment (**a** and **b**) and post-treatment (**c** and **d**), respectively, according to the established cut-offs.

The cut-points for RFS and OS were 1114.0 and 918.5 pg/ml, separating the patients in a group of 10 (27%) above cut-off and 27 (73%) below cut-off in RFS analysis, and a group of 13 (35.1%) above and 24 (64.9%) below cut-off in OS analysis, Fig. 2A,B. There was no significant association between pre-treatment plasma uPAR and any of the outcome measures RFS and OS in Kaplan–Meier analysis (log-rank p = 0.21 and p = 0.3, n = 37).

In univariate Cox analysis, pretreatment plasma uPAR was not predictive of RFS or OS (HR = 3.185 (95% CI 0.403–25.158), p = 0.272 and HR = 2.048 (95% CI 0.510–8.231), p = 0.312) and therefore no multivariate analysis was performed. Pretreatment, high TNM Stage (S3–4) was borderline significant predictive of RFS (HR = 3.089 (95% CI 1.032–9.247), p = 0.044), whereas p16 status significantly predicted OS (HR = 4.257 (05% CI 1.128–16.064), p = 0.033), Table 3.

**Table 3:** Prognostic value of pre-treatment plasma uPAR. Cox proportional hazards model for relapse-free survival (RFS) and overall survival (OS) in relation to clinicopathological variables and pre-treatment plasma uPAR (uPAR-pre) dichotomized according to the established cut-off.

Pre-treatment		RFS			OS		
Variable	n	Univariate analysis			Univariate analysis		
		HR	95% CI	p-value	HR	95% CI	p-value
**Gender**							
Women	7						
Men	42	2.408	0.314–18.448	0.398	2.009	0.257–15.702	0.506
Age*		1.028	0.975–1.085	0.302	1.021	0.961–1.084	0.505
**Smoking**							
< 30 pack years	29						
> 30 pack years	20	1.189	0.416–3.403	0.747	2.275	0.666–7.775	0.190
**T site**							
Pharynx	43						
Non-pharynx	6	1.374	0.725–2.604	0.330	1.184	0.255–5.497	0.829
**T stage**							
T1–2	28						
T3–4	21	1.555	0.545–4.437	0.410	2.950	0.860–10.117	0.085
**N stage**							
N0	14						
N+	35	1.125	0.351–3.605	0.843	1.823	0.556–5.978	0.322
**TNM stage**							
S1–2	28						
S3–4	21	3.089	1.032–9.247	**0.044**	3.152	0.920–10.801	0.068
**p16**							
Positive	29						
Negative	20	2.057	0.713–5.935	0.182	4.257	1.128–16.064	**0.033**
**uPAR-pre**							
< Cutoff	27						
> Cutoff	10	3.185	0.403–25.158	0.272	2.048	0.510–8.231	0.312

Significant values are in [bold].

*Age was included as a continuous covariate. T tumor; N node; M metastasis. Hazards ratio (HR), 95% confidence intervals (95% CI).

#### Post-treatment samples (2 months)

The cut-points for RFS and OS were 886.6 and 994.2 pg/ml, separating a group of 15 (40.5%) above cut-off and 22 (59.5%) below cut-off in RFS analysis, and a group of 10 (27%) above cut-off and 27 (73%) below cut-off in OS analysis, Fig. 2C,D.

Kaplan–Meier curves combined with log-rank analysis for differences showed a significant association between high plasma uPAR post-treatment (2 months) and poor RFS (log-rank = 0.0048) and borderline association with poor OS (log-rank p = 0.055), (n = 37).

In univariate Cox analysis of post-treatment samples, a high post-treatment plasma uPAR significantly predicted RFS (HR = 6.674 (95% CI 1.439–30.947), p = 0.015) but was not associated with OS (HR = 3.288 (95% CI 0.882–12.259), p = 0.076). High TNM stage (S-3–4) was borderline associated with RFS (HR = 3.059 (95% CI 1.021–9.163), p = 0.046) but not with OS (HR = 2.778 (95% CI 0.782–9.869), p = 0.114). In multivariate Cox analysis, including 2-months post-treatment plasma uPAR and TNM stage, only high post-treatment uPAR remained significantly associated with RFS (HR = 6.623 (95% CI 1.399–31.35), p = 0.017), but not with OS (1.000 (95% CI 0.999–1.002), p = 0.704), Table 4.

**Table 4:** Prognostic value of post-treatment plasma uPAR. Cox proportional hazards model for relapse-free survival (RFS) and overall survival (OS) in relation to clinicopathological variables and post-treatment plasma uPAR (uPAR-post) dichotomized according to the established cut-off.

Post-treatment		RFS						OS					
Variable	N	Univariate analysis			Multivariate analysis			Univariate analysis			Multivariate analysis		
		HR	95% CI	p-value	HR	95% CI	p-value	HR	95% CI	p-value	HR	95% CI	p-value
**Gender**													
Women	7												
Men	42	2.40	0.31–18.4	0.400				1.84	0.23–14.5	0.563			
Age*		1.03	0.97–1.09	0.316				1.02	0.96–1.09	0.478			
**Smoking**													
< 30 pack years	29												
> 30 pack years	20	1.18	0.41–3.36	0.762				1.93	0.54–6.84	0.309			
**T site**													
Pharynx	43												
Non-pharynx	6	1.38	0.73–2.61	0.326				1.30	0.28–6.13	0.742			
**T stage**													
T1–2	28												
T3–4	21	1.54	0.54–4.49	0.421				2.58	0.73–9.19	0.143			
**N stage**													
N0	14												
N+	35	1.14	0.36–3.66	0.824				1.44	0.41–5.10	0.573			
**TNM stage**													
S1–2	28												
S3–4	21	3.06	1.02–9.16	**0.046**	1.84	0.51–6.41	0.364	2.78	0.78–9.87	0.114	1.69	0.45–6.30	0.437
**p16**													
Positive	29												
Negative	20	2.03	0.71–5.87	0.189				3.756	0.97–14.6	0.055			
**uPAR-post**													
< Cutoff	22												
> Cutoff	15	6.67	1.44–31.0	**0.015**	6.62	1.40–31.4	**0.017**	3.29	0.88–12.3	0.076	1.00	1.00–1.002	0.704
**uPAR-post**													
< Median	19												
> Median	18	4.86	1.05–22.6	**0.044**	5.143	1.10–24.05	**0.037**	1.11	0.30–4.14	0.875	1.70	0.45–6.37	0.435

Significant values are in [bold].

*Age was included as a continuous covariate. T tumor; N node; M metastasis. Hazards ratio (HR), 95% confidence intervals (95% CI).

The area under the ROC curve (AUC) was 0.728 (0.539–0.919), p = 0.030 2-months post-treatment using the optimal cut-off.

Using the median post-treatment plasma uPAR as cut-off, a high plasma uPAR also significantly predicted RFS (HR = 4.853 (95% CI 1.045–22.546), p = 0.044), but was not associated with OS (HR = 1.11 (95% CI 0.30–4.14), p = 0.875). In multivariate analysis a high post-treatment plasma uPAR remained significant for RFS (HR = 5.143 (95% CI 1.10–24.05), p = 0.037), but not regarding OS. TNM stage was not significant in multivariate analysis regarding RFS nor OS, similar to the analysis including the optimal cut-off.

#### Concordance of plasma uPAR and primary tumor uPAR-PET/CT

Among the patients who participated in both the biobank and the prospective study investigating the prognostic value of uPAR-PET/CT 26 patients donated a sample pre-treatment and 24 patients a 2-months post-treatment sample.

There was no significant correlation between pre-treatment plasma uPAR and primary tumor uPAR expression determined by uPAR-PET/CT SUVmax (B = 133.32 (95% CI − 44.48 to 311.13), p = 0.136).

**Figure 3:**
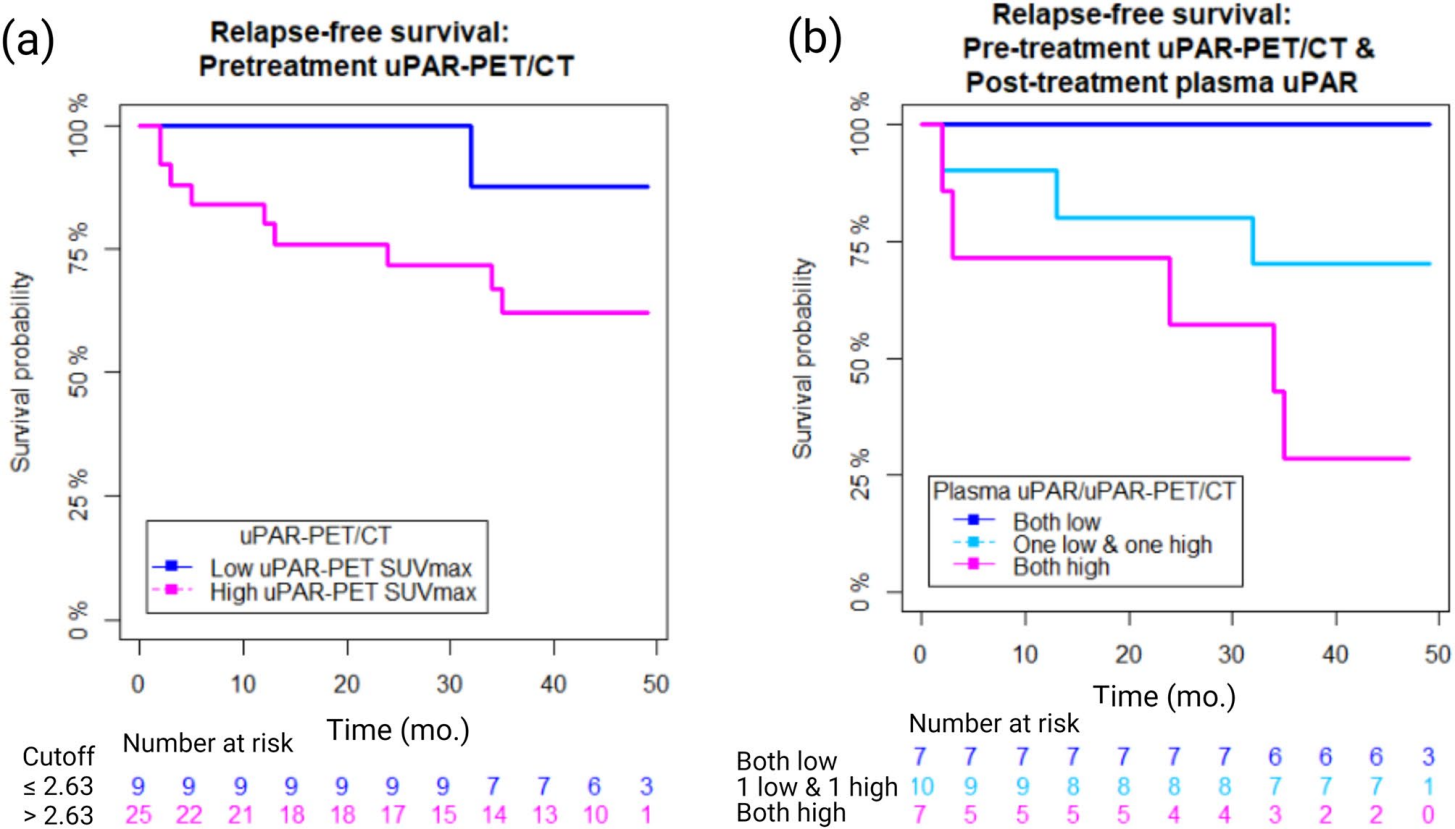
Relapse-free survival according to (**a**) pre-treatment uPAR-PET alone and (**b**) pretreatment uPAR-PET in combination with post-treatment plasma uPAR results in head and neck squamous cell carcinoma patients following radiotherapy treatment. (A) dark blue indicates an SUVmax below cut-off and magenta an SUVmax above cut-off. (B) Congruent low uPAR measurements (dark blue), congruent high uPAR measurements (magenta) and incongruent uPAR measurements (turquoise), with one low and one high estimate on either of the modalities. The uPAR levels determined by liquid biopsies and uPAR-PET were dichotomized according to the established cut-offs.

Using the cut-off established for the prediction of RFS with uPAR-PET/CT and the plasma uPAR cut-off established for RFS at the 2 months control the patients were divided into three groups: (A) patients with both low uPAR-PET/CT pretreatment and low plasma uPAR post-treatment (both low), (B) patients with either low uPAR-PET/CT and high plasma-uPAR or high uPAR-PET/CT and low plasma-uPAR post-treatment (one low/one high) and finally (C) both high uPAR-PET/CT pretreatment and high plasma-uPAR post-treatment (both high), Fig. 3. The concordance rate was 58.4% (both low or both high) and the discordant rate was 41.6% (one low/one high). The Kaplan–Meier curve is presented in Fig. 3. Overall, there was a significant difference between the groups (log-rank p = 0.025). Patients with both a high uPAR-PET/CT SUVmax and high plasma uPAR post-treatment (both high group, n = 7) had a significantly reduced RFS compared to patients with low uPAR-PET/CT and low plasma uPAR post-treatment (both low, n = 7), (log-rank p = 0.008). Although non-significant (NS), patients with incongruent results (one low/one high, n = 10) showed a trend of having a favorable prognosis compared to patients with high results from both modalities (log-rank p = 0.131), but inferior compared to patients with low uPAR-estimates on both modalities (p = 0.136).

## Discussion

The main finding of the current study was the inability of pre-treatment plasma uPAR to predict RFS and OS in HNSCC patients referred to curatively intended radiotherapy, in contrast to uPAR-PET/CT. However, plasma uPAR measured at the 2-months follow-up predicted long-term RFS. In a multivariate model including post-treatment plasma uPAR, together with the most validated and clinically established predictor of survival outcome, TNM-stage, only post-treatment plasma uPAR remained significant for RFS.

Total plasma uPAR is known to originate from many non-cancerous biological processes (e.g. infection, liver cirrhosis and particularly alcohol induced liver disease etc.) and a considerable overlap in plasma uPAR has been described in gynecological cancers and controls^15,16^. The potential false positive non-tumor-derived increase in total plasma uPAR and the multiple competing alcohol- and tobacco-associated diseases in HNSCC patients can therefore obscure the individual prediction of HNSCC relapse.

Furthermore, total plasma uPAR levels may be similar from a low uPAR expressing large tumor volume and a high uPAR expressing small tumor volume. Finally, the proportion of the cell bound uPAR that is released may depend on several non-cancerous factors. This may explain why the current study, and similar studies, were not able to show an association between the pre-treatment total plasma uPAR and the clinicopathological characteristics like T-, N- and TNM stage^13^.

Regarding treatment stratification, clinicians need a tumor specific pre-treatment biomarker to predict the risk of recurrence. However, in our study pre-treatment total plasma uPAR was not able to predict RFS, in line with the results of previous studies, and a post-treatment sample is irrelevant for selecting therapy^8^. In contrast, we have previously shown that uPAR-PET/CT predicted RFS in a similarly sized study (n = 54)^17^. The advantage of uPAR-PET compared to measurement of total plasma uPAR is that it is not susceptible to non-cancerous origin of uPAR, since the measurement is limited to the tumor volume on PET/CT. Additionally, PET circumvents sampling error compared to traditional biopsy. Cancer aggressiveness is probably best related to the maximal local expression of uPAR as the most aggressive phenotype will determine the prognosis and traditional biopsy may over- or underestimate the aggressiveness depending on the sampling site. This strongly supports the idea of using an image-derived and tumor-specific “in-situ” measurement of the heterogoenous uPAR expression for treatment stratification, which is exactly what uPAR-PET is capable of doing as demonstrated in our previous study^17^.

In general, biomarkers are assessed at baseline, i.e., prior to treatment initiation, to estimate the predictive value. However, baseline predictions become weaker with time and more recent biomarker values may better reflect the current status of the patient after treatment if e.g. concomitant chemotherapy or radio sensitizing treatment was discontinued^18^. Consequently, since post-treatment plasma uPAR was able to predict RFS a routine plasma uPAR measurement at the 2-monhts control may offer an opportunity to support clinicians in tailoring of individual risk stratified follow-up programs in the future.

As our results suggest, patients with high uPAR-estimates on both the pre-treatment uPAR-PET/CT and the 2-months post-treatment plasma sample could be offered a more intensive surveillance program, whereas patients with low uPAR levels on both modalities could be referred to a less extensive follow-up program, since the patients often request a less frequent surveillance program^19^. Additionally, as HNSCC patients frequently develop new primary cancers, subsequent plasma uPAR liquid biopsies may improve the detection of patients with subclinical new primary cancers or recurrences.

The prognostic value of increasing uPAR levels in blood and tissue samples is well-documented and reflects an imbalance of the urokinase proteolytic system, which results in an overall higher proteolytic activity for the cancer cells to break down the ECM and cause invasion and metastasis^5–9,20^. During radiotherapy treatment our results demonstrated an increase in plasma uPAR. Similarly, an increase in uPAR expression has been documented by other studies, e.g. a study analyzing plasma samples from esophageal cancer patients undergoing radiotherapy and a study on tissue samples collected from p16-positive cervical cancer patients also undergoing radiotherapy^7,8^. In contrast, a small study on HNSCC patients showed a reduction in plasma uPAR (n = 11), but the patients were referred to surgery and the timing of the samples were varying (1–42 days post-treatment)^13^.

Studies have demonstrated a reduced proliferation, but interestingly also a simultaneous enhanced cell adhesion, migration and invasion of cancer cells following sublethal doses of radiation^10^. Additionally, an in vitro study on medulloblastoma cells indicated that the radiation-induced invasion was mediated by an increased uPAR expression^10^. Furthermore, the authors demonstrated that the radiation induced invasiveness could be inhibited by targeting- and thereby down-regulating uPAR expression prior to radiotherapy. The molecular mechanism is not yet understood, but a high post-treatment plasma uPAR may indicate that the radiotherapy had insufficient therapeutic efficacy and that uPAR may offer a future prognostic biomarker and potential target for cancer treatment.

uPAR has also been suggested to be upregulated by radiation-induced inflammation and hypoxia^21^. Alternative theories suggest that uPAR is released into the blood stream by necrotic cancer cells or it could reflect tumor vascularization^4,13^. These mechanisms could be interesting to investigate in future studies.

In line with the literature, the current results demonstrate a considerable overlap of the uPAR level in plasma from controls and HNSCC patients^9^. Our study has limitations including the size of the biobank and the matching of controls regarding smoking, performance status (PS) and gender. Since plasma uPAR has been associated with gender and smoking, correct matching of controls is important. Unfortunately, due to the COVID-19 pandemic recruiting controls with a considerable smoking history and poor performance status was unethical and unfeasible. Instead, age-matched hospital employees were recruited, although a larger proportion were female non-smokers with good performance status.

We did not find a significant association between smoking history and plasma uPAR among the controls. The power may have been too low to detect a possible association among the controls (n = 19), since there was a significant association among the patients (n = 49). Moreover, the patients had a relatively good PS and therefore a large proportion tolerated nimorazole and had a better prognosis compared to the general population with HNSCC. In larger future studies inclusion of more patients with poor PS is needed to generalize the results to the HNSCC population and the optimal cut-off may change.

It has been argued that optimal cut-off determination can lead to peculiar group sizes and false discoveries. However, both the optimal cut-off and the median post-treatment uPAR significantly predicted RFS in the current study. The non-significant prediction of OS may indicate that the follow-up was too short to draw firm conclusions. Inclusion of more patients with a poor prognosis a priori regarding PS, smoking etc. may increase the statistical power due to more events and clarify the prognostic impact of plasma uPAR regarding OS. Finally, since p16 positive patients have a superior prognosis, larger studies are needed to explore the prognostic value in the p16 negative and positive subgroup separately.

In summary, pre-treatment uPAR-PET/CT offers an interesting opportunity for stratification of the treatment to HNSCC patients according to individual risk assessments and in combination with post-treatment plasma uPAR the surveillance program could be optimized further in respect to updated liquid biopsies evaluating the effect of the treatment. Larger prospective trials are needed to validate the results of the current explorative study and allow for inclusion of more factors in a multivariate analysis, e.g. smoking, gender, age, performance status and nimorazole treatment. However, we believe our results represent an interesting first step towards improving future management of HNSCC patients.

## Conclusion

Pre-treatment plasma uPAR did not predict RFS nor OS in HNSCC patients referred to curatively intended radiotherapy, in contrast to pre-treatment uPAR-PET/CT, which has previously been shown to predict RFS. However, plasma uPAR measured at the 2 months follow-up predicted long-term RFS. In a multivariate model including the post-treatment plasma uPAR measurement together with TNM stage, only post-treatment plasma uPAR remained significant for RFS.

Accordingly, pre-treatment uPAR-PET/CT and not plasma uPAR offers a potential tool for treatment stratification, whereas post-treatment plasma uPAR may assist clinicians in tailoring post-treatment follow-up programs according to the individual risk assessment.

## Material and methods

### Study population

The inclusion criteria of the biobank were patients above 18 years with newly diagnosed biopsy-verified cancer of the pharynx, larynx or oral cavity, who were referred to curatively intended radiotherapy at Rigshospitalet or Næstved Hospital, Copenhagen, Denmark, between September 1st 2017 and November 30th 2021. In parallel, 19 healthy controls aged 55–85 years were included.

Exclusion criteria were synchronous cancers or former HNSCC within 5 years, blood transfusion within 3 months, or chronic inflammatory- or hematological diseases.

The study was performed in accordance with the Declaration of Helsinki regarding regulations on research involving human participants. The participants provided written informed consent to donate blood to a research biobank in future research in cancer and/or genetic research under an existing protocol at the Danish Capital Region’s Committee on Health Research Ethics (Protocol number H-16039798). The current study using the blood samples from the biobank and blood samples from 19 age-matched controls was approved by the Capital Region’s ethics committee (H-19053309). Signed informed consent was obtained from all controls, who were recruited by posters at the hospital.

Twenty-six of the patients had a uPAR-PET/CT scan performed pretreatment at the time of the pre-treatment blood sample as a part of another study protocol approved by the same Capital Regional Committee (H-16039798; EudraCT no. 2016-002082-65)^14^.

### Clinical data collection

All baseline data were collected before the initiation of radiotherapy. Diagnosis of HNSCC was verified histologically prior to inclusion. Information on alcohol, smoking, p16 status, clinical examination, treatment plan, medical history, laboratory- and histological results, and follow-up examinations were collected from patient records. Disease stage was coded according to Union for International Cancer Control (UICC) 8th edition^22^.

According to national guidelines all head and neck cancer patients attended a 5-year follow-up program including a clinical examination 2 and 6 months after radiotherapy, every 6 months for 2 years and every 12 months for the last 3 years, for a total of 5 years. Histological verification of loco-regional recurrences was obtained in all patients serving as reference for the study outcome.

### Sample preparation

Originally, Streck tubes are blood collection tubes available for stabilizing cell-free DNA and RNA. (Supplier: Nordic BioSite ApS, Copenhagen, Denmark). The specialized preservative limits cell lysis and conserve the blood for 14 days at room temperature, which preserves sample integrity during storage, shipping, and handling of blood samples^23^.

#### Patients

All blood samples obtained before treatment were collected at the Capital hospital (Rigshospitalet) and processed within 30–45 min. All post-treatment samples were stored at room temperature until centrifugation at Rigshospitalet within 24 h (two samples after 3 days). The samples were collected during day time. All blood samples were centrifuged at 1600×*g* for 10 min at room temperature (21 degrees Celsius). Subsequently, the supernatant was transferred to 1.5 ml PCR clean Eppendorf tubes, recentrifuged at 16,000×*g* and stored at − 80 °C after removal of debris.

#### Healthy controls

Peripheral blood samples from 19 age-matched controls were collected and processed as the patient samples in 9 ml Streck tubes. Baseline information on smoking history, age and medical history was registered as well.

### Bead-based immunoassay

The total uPAR protein level was measured in plasma samples using a human bead-based immunoassay (Procartaplex, Thermofisher, Roskilde, DK) and analyzed according to the protocol of the manufacturer. Briefly, samples were thawed, vortexed for 30 s and centrifuged for 10 min at 3000*g*, following which 25 μl of sample was mixed with magnetic beads and incubated for 2 h. Then, antibody-mix was added, and plates incubated for 30 min following which Streptavidin-PE was added and plates incubated for 30 min. Reading buffer was added and plates read on the Luminex 200 instrument (Luminex, Austin, Texas). Incubations were done at room temperature upon shaking and all steps were preceded by 2 rounds of washing. A standard curve (in duplicate) was included on all plates (7 dilutions, fourfold dilution), samples were run in simplex and concentration calculated from the standard curve in pg/ml.

### Statistical analysis

The Wilcoxon paired samples test was performed to compare the plasma concentrations of uPAR in the individual patients pre- and post-treatment. The independent samples Mann–Whitney U-test was used to compare the plasma concentration of uPAR in the patients pre- and post-treatment to the controls and to assess the association of the pre-treatment uPAR concentration to the clinicopathological characteristics p16-status, T- and N-stage, age (dichotomized as beneath or above 66 years), smoking history (dichotomized as less or more than 30 pack years) and gender.

In survival analysis, the clinical endpoints were relapse-free survival (RFS) defined as time from diagnosis to any relapse of the disease at the locoregional site and/or distant metastasis with deaths from other causes recorded as censoring. Overall survival (OS) was defined as time from diagnosis to death of any cause. For the pre- and post-treatment samples the survival time was calculated from the day the blood sample was collected. Determination of the optimal cut-off in discrimination between good and poor prognosis was performed with ROC curve analysis and Cut-off finder, an R-package developed by Budczies et al.^24^. Association between the biomarker concentration beneath and above cut-off and survival outcomes were visualized in Kaplan–Meier plots using log-rank test to assess the significance of the difference. Hazard ratios were estimated in univariate and multivariate Cox proportional hazards model in which the uPAR concentration was dichotomized according to the defined cut-offs for RFS and OS for both the pre- and post-treatment samples.

The number of events included in the survival analysis were: 14 events in RFS analysis and 11 events in OS analysis. Based on the number of events only two explanatory predictors could reasonably be included in the multivariable analysis. In addition to plasma uPAR, TNM stage was included as it is the most important clinically established predictor of survival.

In post-hoc analysis, the association between the continuous covariates plasma uPAR and uPAR SUVmax were tested in a general linear model.

P-values < 0.05 were considered statistically significant. All statistical analyses were performed using IBM SPSS Statistics v. 25 (IBM Corp. Armonk, NY) and R project for Statistical Computing, 2021.

## Data Availability

The data that support the findings of this study are available from the corresponding author upon reasonable request.

## Abbreviations

^68^Ga: Gallium-68
AE105: Ac-Asp-Cha-Phe-(D)Ser-(D)Arg-Tyr-Leu-Trp-Ser-CONH2
HNSCC: Head and neck squamous cell carcinoma
NOTA: 2,2′,2″-(1,4,7-Triazacyclononane-1,4,7-triyl)triacetic acid
OS: Overall survival
RFS: Relapse-free survival
