# Author Correction: Urokinase-type plasminogen activator receptor (uPAR) assessed by liquid biopsies and PET/CT for prognostication in head and neck cancer patients

**DOI:** 10.1038/s41598-023-28618-9

**Published:** 2023-01-24

**Authors:** Louise Madeleine Risør, Tina Binderup, Marie Øbro Fosbøl, Kim Francis Andersen, Annika Loft, Jeppe Friborg, Andreas Kjaer

**Affiliations:** 1 Department of Clinical Physiology and Nuclear Medicine & Cluster for Molecular Imaging, Copenhagen University Hospital, Rigshospitalet, Blegdamsvej 9, 2100 Copenhagen, Denmark; 2 Department of Biomedical Sciences, University of Copenhagen, Copenhagen, Denmark; 3 Department of Clinical Oncology, Copenhagen University Hospital, Rigshospitalet, Copenhagen, Denmark

Correction to: *Scientific Reports* https://doi.org/10.1038/s41598-022-21175-7, published online 09 November 2022

Kim Francis Andersen was inadvertently omitted from the author list in the original version of this Article.

The Author Contributions section now reads:

“L.M.R. and A.K. conceived the study and design. L.M. included the patients and drafted the manuscript; T.B. performed the experiments; M.F., K.F.A., A.L., J.F. and A.K. supported the data analysis and critically revised the manuscript. All authors read and approved the final manuscript.”

The original Article has been corrected.

